# Microvessel density in head and neck squamous cell carcinoma

**DOI:** 10.1007/s00405-018-4996-2

**Published:** 2018-05-10

**Authors:** Tomasz Szafarowski, Janusz Sierdzinski, Miroslaw J. Szczepanski, Theresa L. Whiteside, Nils Ludwig, Antoni Krzeski

**Affiliations:** 1 Department of Otolaryngology, Faculty of Medicine and Dentistry, Czerniakowski Hospital, Medical University of Warsaw, 19/25 Stępińska Str., 00-739 Warsaw, Poland; 2 Department of Medical Informatics and Telemedicine, Medical University of Warsaw, Banacha 1a Str., 02-097 Warsaw, Poland; 3 Department of Biochemistry and Clinical Chemistry, Faculty of Pharmacy, Medical University of Warsaw, Banacha 1a Str., 02-097 Warsaw, Poland; 4 Laboratory of Centre for Preclinical Research, Medical University of Warsaw, Banacha 1a Str., 02-097 Warsaw, Poland; 5 Department of Pathology, University of Pittsburgh School of Medicine, Pittsburgh, PA 15213 USA; 6 Departments of Immunology and Otolaryngology, University of Pittsburgh School of Medicine, Pittsburgh, PA 15213 USA; 7 UPMC Hillman Cancer Center, Pittsburgh, PA 15213 USA

**Keywords:** Head and neck squamous cell carcinoma, Larynx dysplasia, Microvessel density, CD34, CD105, Tissue microarrays

## Abstract

**Purpose:**

Microvessel density (MVD) corresponds to the intensity of neo-angiogenesis. MVD assessments are based on the expression levels of the vascular endothelium markers such as, e.g., CD34 or CD105. The goal of this study was to assess MVD among patients with head and neck squamous cell carcinoma (HNSCC), and to evaluate the predictive value of MVD in head and neck cancers.

**Methods:**

The study included 49 patients treated for HNSCC and 11 patients with dysplasia of the upper respiratory tract epithelium. Control tissues consisted of 12 normal mucous membranes of the throat. Expression levels of MVD markers were assessed by immunohistochemistry (IHC) using tissue microarrays (TMA). Clinicopathological factors and patients’ survival over the 5-year follow-up period were analyzed.

**Results:**

The MVD/CD34 values were found to be significantly elevated in the HNSCCs compared to the non-malignant control tissues (*p* = 0.001) and to dysplastic tissues. (*p* = 0.02). Significantly higher MVD/CD105 values were also seen in the tumor compared to the control tissues (*p* = 0.001) or the dysplastic tissues (*p* = 0.001). Unexpectedly, significantly lower MVD/CD34 values were seen in the tumor tissues of patients with the T3–T4 tumors compared to those with T1–T2 tumors (*p* = 0.01).

**Conclusions:**

HNSCCs have statistically higher MVD values compared to dysplasia of the upper respiratory tract epithelium. However, the MVD/CD34 values did not correlate with local invasiveness (the T feature) of HNSCCs. This counterintuitive observation suggests that assessments of MVD as performed on TMA by IHC using anti-CD34 or anti-CD105 antibodies considered to be specific for endothelial cell markers might underestimate the extent of the tumor vascularity in HNSCC.

## Introduction

Squamous cell carcinomas of the head and neck (HNSCC) remains a significant therapeutic challenge, and the effectiveness of current treatments remains the same as it was 40 years ago [1]. According to a concept put forward by Folkman in 1971, tumor growth depends on local neo-angiogenesis [2]. Assessments of angiogenesis in tumors, including HNSCCs, require application of reliable indicators, or markers of angiogenesis, which may be measured using methods such as immunohistochemistry (IHC) with antibodies specific for antigens present on the vascular endothelium, including CD34 or CD105. As proposed by Weidner et al., microvessel density (MCD) is calculated as a mean number of microvessels identified using a light microscope with the known field diameter. These investigators were the first to report an association between MVD and prognosis in breast cancers [3]. This report generated much interest and initiated a search for similar associations in a variety of other neoplasms in hope of identifying new prognostic factors.

CD34 is a membrane glycoprotein found on the external surface of endothelial cells. During angiogenesis, the CD34 protein is responsible for adhesion of leukocytes to the internal surface of a vascular wall and for migration of vascular endothelial cells [4]. CD34 is expressed in all endothelial cells during proliferation as well as in the G_0_ stage, and anti-CD34 Abs react with the largest number of endothelial cells compared to other antibodies used in studies of tumor angiogenesis. However, anti-CD34 Abs may also interact with various elements of the matrix, limiting their specificity in MVD assessments [5].

The CD105 antigen (endoglin) is a homodimeric membrane glycoprotein consisting of two subunits connected by a disulfide bridge. CD105 is a component of the TGF-ß receptor complex [6]. CD105 is present mainly on the f vascular endothelium, but may also be also found on the surface of hematopoietic cells and fibroblasts [7]. CD105 glycoprotein is exclusively expressed on actively proliferating endothelial cells, where endoglin is involved in the control of cell proliferation, migration, and formation of new vessels [7]. The exact role of endoglin in angiogenesis remains unclear, although it has been demonstrated that the presence of endoglin in the tumor vascular endothelium is a negative predictive factor in various types of solid tumors [8].

The reports of MVD in head and neck cancers show high levels of variability, which might reflect a diversity in detection methodologies used in the previous studies. The goal of this work was to evaluate MVD in HNSCCs, using tissue microarrays (TMA) to ensure the uniform tissue quality for a reliable MVD measurements and to assess the relationship between the MVD values and selected clinical and pathomorphological features of the disease. These data were expected to indicate whether MVD plays a role in HNSCC as a potential predictive marker of survival.

## Materials and methods

A retrospective analysis of patients with primary HNSCC (*n* = 49) and patients with dysplasia of the endothelium in the upper respiratory tract (*n* = 11) was conducted. Control group consisted of mucus membranes acquired from patients undergoing soft palate surgery due to obstructive sleep apnea (OSA) (*n* = 12). All patients were treated at the Department of Otolaryngology of the Faculty of Medicine and Dentistry at the Medical University of Warsaw between 2007 and 2011. The study was approved by the Ethics Committee at the Medical University of Warsaw, and all patients signed informed consent forms (AKBE/50/12# to T.S.). Clinicopathological assessment was conducted based on the TNM and UICC classifications. Only patients fulfilling the requirement for 5-year follow-up were included in this study (Table 1).

**Table 1:** Clinicopathological characteristics of the HNSCC patients with primary tumors as well as genders and ages of healthy controls included in this study

Characteristics	HNSCC patients		Dysplasia patients		Healthy controls	
	*n* = 49	(%)	*n* = 11	(%)	*n* = 12	(%)
Sex						
Male	45	92	9	82	12	100
Female	4	8	2	18	–	
Age						
Average ± SD	60.3 ± 8.81		61.8 ± 6.25		55.4 ± 8.98	
Median	60.00		58.50		63.00	
Tumor site						
Larynx and hypopharynx	38	78				
Oral cavity and tongue	11	22				
Clinicopathological characteristics						
Tumor size						
T1	4	8				
T2	9	18				
T3	19	39				
T4	17	35				
Tumor differentiation						
G1	2	4				
G2	39	80				
G3	8	16				
Nodal involvement						
N+	19	39				
N−	30	61				
Nodal involvement						
N1	7	37				
N2a	4	21				
N2b	7	37				
N2c	1	5				
Treatment						
Tumor resection	20	41				
Tumor resection + neck dissection	29	59				
Stage						
I	2	4				
II	5	10				
III	16	33				
IVa	26	53				

Among the group of patients alive at the time of the analysis, the follow-up period was 60–104 months.

### Tissue microarrays

TMA were prepared using the available stored histopathological samples. Single paraffin blocks containing tissue material obtained from patients with HNSCC were prepared. For each tumor, the material was collected from at least two sites indicated by the pathologist as a neoplastic focus. An individual slice obtained using the TMA method was placed on the microscope slide and colored as a single microscope specimen.

### Immunohistochemistry

To visualize staining, we used the Novolink Polymer Detection System (Novocastra and Dako) according to the producer’s recommendations. To identify the cells of vascular origin, we used the murine monoclonal antibody antiCD34 Class II (clone:QBEnd 10) at 1:50 dilution (Dako); murine monoclonal antibody antiCD105 (clone:SN6h) at 1:25 dilution (Dako). Slices were deparaffinized in xylene, and dehydrated in ethanol solutions and distilled water. They were incubated in the TBS buffer (0.1 mmol/l Tris buffer solution, pH = 7.6) for 10 min at a room temperature (RT). To uncover relevant antigen determinants, slides were incubated with TRS High pH buffer (Target Retrieval Solution, pH 9) (Novocastra). Endogenous peroxidase activity was blocked with a 3% hydrogen peroxide solution (Protein Block by Novolink Polymer Detection System, Novocastra). Incubation with reagents was conducted overnight in a moist chamber of the refrigerator at a 4 °C. Each TMA was covered with 300 µl of antibodies. Negative controls consisted of specimen where IHC Diluent (Novocastra) was used instead of a primary antibody. Positive controls consisted of reactions conducted on tissues recommended by the producer of the antibody.

Specimens were assessed blinded using a light microscope by two independent examiners (T.S. and M.J.S.) and the results were averaged. MVD was calculated as an arithmetic mean of the number of microvessels from three fields of view with the greatest vascularization located on two different casts from the same patient. Under 100× magnification, we chose the sites of the greatest vascularity (hotspots), and individual microvessels were counted under 400× magnification. Each immunopositive structure (round, oval, and irregular) separated from other profiles or tissue elements was counted as a single vessel. Each single colored cell or a group of cells were considered a vessel regardless of the presence of vessel lumen. Vessels with visible muscular layer and morphotic elements apparent in the lumen were not counted as a microvessel. The final result was expressed as a number of vessels in the field of view under 400× magnification. The size of microscopic field was 0.236 mm^2^.

### Statistical analysis

The acquired results were analyzed using SAS 9.4 software. The Chi-square test was used for binary variables. Comparison of continuous variables using Student’s *t* test or Mann–Whitney test depending on their distribution demonstrated differences between groups. Kaplan–Meier analysis was used to draw total survival curves in groups depending on MVD value. Differences in survival were assessed using the log-rank test. Logistic regression model with odds ratios (OR) was used in the analysis of likelihood of survival. The level of significance was set at *p* < 0.05. All values represent means ± SD.

## Results

### The MVD/CD34 and MVD/CD105 values

**Fig. 1:**
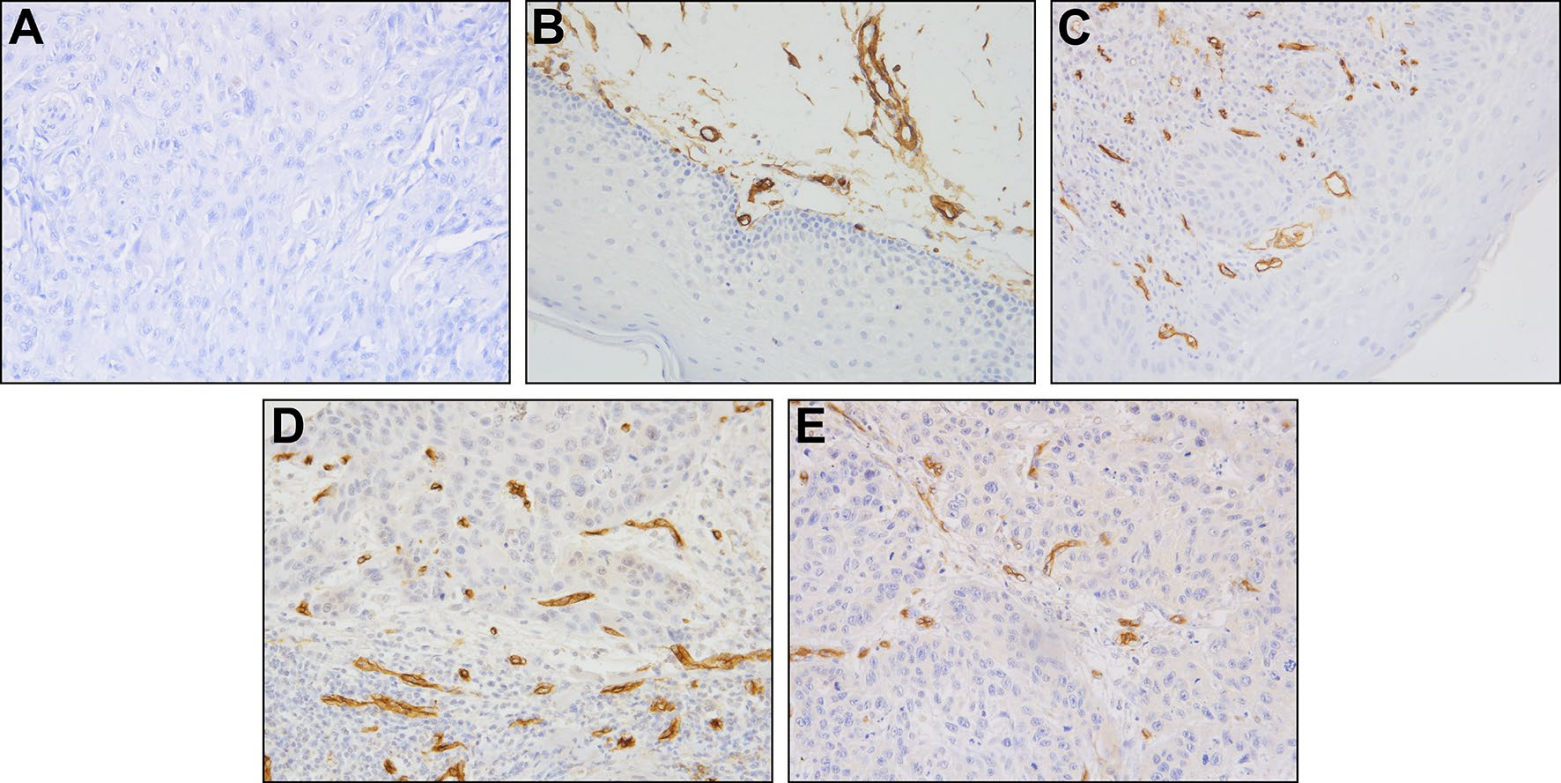
Representative immunohistochemical staining of CD105. Isotype Ab control in specimen of HNSCC (**a**) (× 200), expression of CD105 in specimen of control oral mucosa (**b**) (× 200), expression of CD105 in specimen of dysplastic mucosa (**c**) (× 200), expression of CD105 in specimen of HNSCC (high MVD) (**d**) (× 100), and expression of CD105 in specimen of HNSCC (low MVD) (**e**) (× 200)

Analysis of CD34 expression conducted using Student’s *t* test demonstrated significantly higher mean MVD/CD34 values in the tumor region (21.04 ± 6.47) compared to the normal control group (9.91 ± 1.67), (*t* = 5.87 for *p* = 0.001) and the dysplasia group (16.27 ± 3.25), (*t* = 2.36 for *p* = 0.02). Similar results were obtained for the assessment of endoglin (CD105) expression. Student’s *t* test confirmed a significantly higher mean MVD/CD105 ratio within the tumor (14.04 ± 4.29) compared to the normal control group (2.5 ± 10), (*t* = 17.03 for < 0.001) and the dysplasia group (9.00 ± 1.54), (*t* = 10.65 for *p* < 0.001) (Fig. 1; Tables 2, 3 and 4).

**Table 2:** Results of MVD in the HNSCC group and the healthy controls (Student’s *t* test)

Variables	HNSCC patients		Healthy controls		Test Cochrano–Coxa	*p* value	Confidence interval
	Average	SD	Average	SD			− 95 to 95%
MVD/CD 34	21.04	6.47	9.91	1.67	10.65	< 0.0001	9.03–13.21
MVD/CD105	14.04	4.29	2.5	1.00	17.03	< 0.0001	10.18–12.89

**Table 3:** Results of MVD in the HNSCC group and the dysplasia patients (Student’s *t* test)

Variables	HNSCC patients		Dysplasia patients		Test Cochrano–Coxa	*p* value	Confidence interval
	Average	SD	Average	SD			− 95 to 95%
MVD/CD 34	21.04	6.47	16.27	3.26	3.53	< 0.0001	2.01–7.52
MVD/CD105	14.04	4.29	9.00	1.55	6.54	< 0.0001	3.49–6.59

**Table 4:** Results of MVD in the dysplasia patients and the healthy controls (Student’s *t* test**)**

Variables	Healthy controls		Dysplasia patients		Test Cochrano–Coxa	*p* value	Confidence interval
	Average	SD	Average	SD			− 95 to 95%
MVD/CD 34	9.92	1.67	16.27	3.26	− 5.80	< 0.0001	8.85–10.98
MVD/CD105	2.50	1.00	9.00	1.55	− 11.84	< 0.0001	14.08–18.46

### Analysis of significance of differences between patient groups

**Fig. 2:**
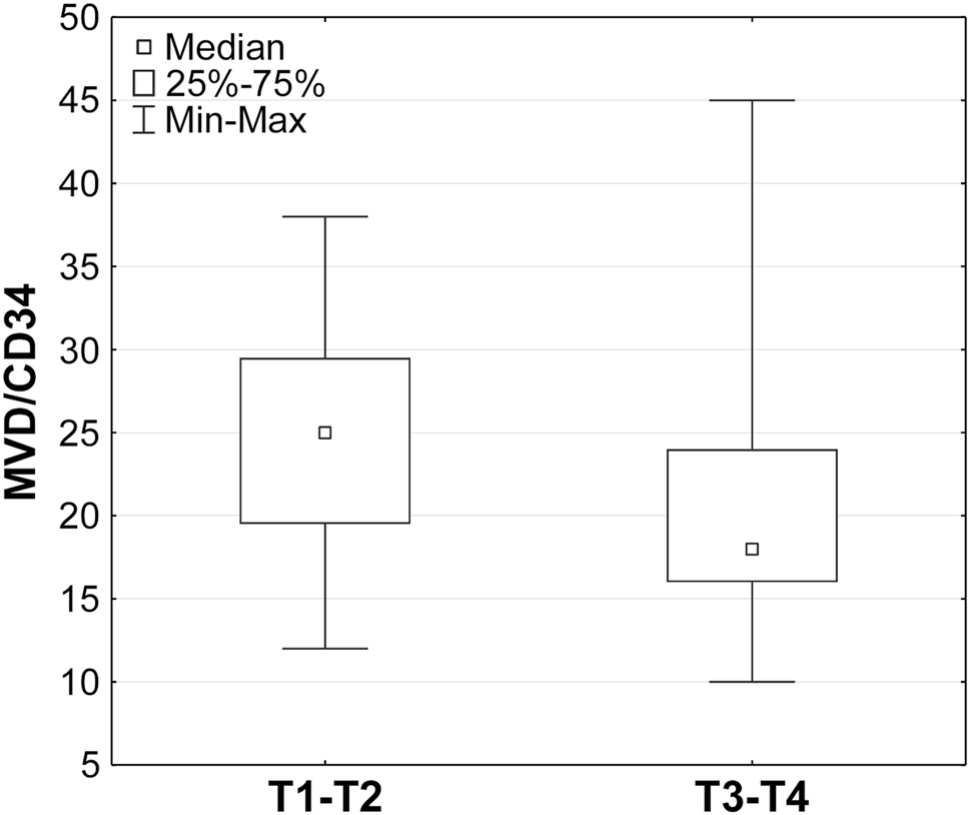
MVD/CD34 vs. tumor size. Comparison of patients with tumors T1–T2 vs. patients with T3–T4 tumors (Mann–Whitney test; *p* = 0,01). Box and whisker plot showing median, range of 25–75% and the min–max values

**Fig. 3:**
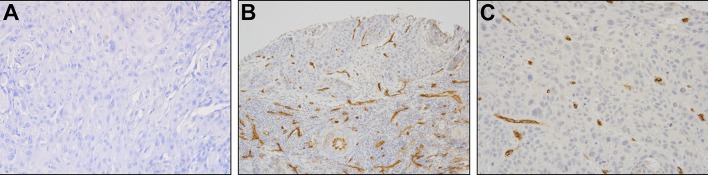
Representative immunohistochemical staining of CD34. Isotype Ab control in specimen of HNSCC (**a**), expression of CD34 (high MVD) in T1 tumor in specimen of HNSCC (**b**), and expression of CD34 (low MVD) in T4 tumor in specimen of HNSCC (**c**); (× 200)

The Mann–Whitney test demonstrated significantly lower MVD/CD34 ratios among patients with T3–T4 tumors compared to those with T1–T2 cancers (*U* = 442 for *p* = 0.01). This was an unexpected finding. However, no such relationship was found for the MVD/CD105 values and the demographic, clinical, and histopathological characteristics, such as age, sex, and histopathological grading (G feature) of the HNSCC patients, as shown in Figs. 2 and 3.

### Analysis of postoperative patient survival

Five-year survival in the patients group was 45%. Taking into consideration the following characteristics: age, sex, histopathological grading, tumor size (T feature), clinical staging (TNM), MVD/CD34, and MVD/CD105, we built a multivariate logistic regression model containing characteristics considered significant for patient survival. No characteristics influencing probability of patient survival were identified. Kaplan–Meier analysis was used to obtain total survival curves for the patient groups divided based on the MVD values, and no differences in 5-year survival were found in relation to the MVD values.

## Discussion

Remodeling of the existing vessels and formation of new ones is the key component of the neoplastic transformation, enabling the tumor to grow. Density of microvessels (MVD) reflects the intensity of angiogenesis within the tumor. In 1991, Weidner et al. proposed the MVD assessment as one of the measures of tumor vascularity [9]. From the moment, Weidner et al. described a relationship between lymph node metastasis and MVD in breast cancer; many researchers have attempted to correlate MVD values with clinical and pathological factors in various types of neoplasms. A relationship was demonstrated between higher MVD and prognosis in non-small cell lung carcinoma, breast, and colon cancers [10–12]. However, some of the studies failed to show that MVD is a prognostic factor predicting patient survival [13].

Many subsequent studies have used endothelial cell markers such as CD34 or CD105 for the analysis of MVD. In 2002, Schimming and Marme were the first to analyze MVD based on CD105 identification in HNSCC. They demonstrated a correlation between tumor size and the higher MVD/CD105 ratio in oral cancers [14]. Based on this and similar studies, the concept emerged that the high MVD/CD105 values are associated with the presence of lymph node metastasis and poorer 5-year survival [15–19]. This and other studies [20] suggested that CD105 expression might serve as an important prognostic factor in HNSCC, enabling the identification of patients at high risk of recurrence and shorter survival.

In this study, no statistically significant correlations were observed between the MVD values in HNSCCs and the tumor stage or other clinicopathological characteristics. Nor was there a correlation between the MVD values and patients’ survival. This lack of correlations could be attributed to the use of TMAs for the MVD assessment. Since microvascularization differs within the tumor, MVD is usually averaged from several or several dozen places throughout tumor cross-section, whereas the TMA technique allows for microvessel assessment only in its selected fragments. Thus, TMAs might not fully reflect the true degree of microvascularization in a tumor, as also suggested by others [21, 22].

To the best of our knowledge, our study is the first to simultaneously assess MVD/CD105 and MVD/CD34 in HNSCC using TMAs. According to various reports, the results of MVD assessments using CD105 expression in HNSCCs vary over a wide range. In our data set, mean MVD in the study group was 61.34/mm^2^ for HNSCC and 2.61/mm^2^ for normal mucus membranes. Bodnar et al. reported higher MVD values in a group of patients with laryngeal cancers [23]. Zvrko et al. reported results similar to our findings among patients with glottis and epiglottis cancers [24, 25]. However, Martone et al., Shimming et al., and Chien et al. reported lower MVD values [14, 17, 26]. A wide range of values is also reported for the results of MVD assessment based on CD34 expression. In our results, mean MVD/CD34 was 21.46 (90.53 mm^2^) in HNSCC and 10.1 (42.61 mm^2^) in normal mucus membranes. Others have reported variable results [27–30].

Our results suggest that MVD, as currently measured, does not accurately reflect the degree of tumor vascularity. The higher MVD/CD34 values in the T1–T2 compared to T3–T4 cancers are counterintuitive and suggest that the current practice of using CD34 as a marker of MVD may be questioned. CD34 is expressed on hematopoietic cells, including early progenitor cells, and its abundance on microvessels in T1–T2 HNSCCs could be taken as an indication of an early differentiation stage of endothelial cells in the newly forming vessels. On the other hand, anti-CD34 Ab is considered as the “para-endothelial Ab” that reacts with antigens located on the surface of all endothelial cells regardless of their functional status. It was also demonstrated that anti-CD34 Abs react with the largest number of endothelial cells compared to other commonly used Abs [21]. Indeed, in this study, the highest MVD values were obtained by analyzing CD34 expression, and the lowest values were obtained for CD105. It is generally thought that endoglin (CD105) rather than CD34 is expressed in the vascular endothelium of tumor vessels forming de novo [8]. The role of CD34 and endoglin as potential markers of new vessels vs. functioning, mature vessels is currently unclear. Given the different specificities of anti-CD34 and anti-CD105 antibodies for endothelial cells, this is not surprising. Further studies will be necessary to establish which of the two proteins will emerge as a more reliable marker of all vessels and thus a more suitable marker for measurements of MVD in all tumors.

The variability of the MVD data in the literature suggests a need for a closer scrutiny of the reasons behind these discrepancies. The most critical concerns relate to: (a) the disease heterogeneity, emphasized by the fact that HNSCC is a disease encompassing several distinct sites and tumor histologies; (b) the antibodies used for MVD assessments vary in their quality and specificity; (c) methods for the tissue selection and handling differ; and (d) the assessment algorithms used to quantify MVDs are selected using disparate criteria that are based on the experience of individual investigators. There is an unmet need for applying a uniform IHC methodology to MVD assessments, including the use of antibodies that are specific for all endothelial cells and are appropriately used according to the standard experimental protocol. Currently, neither endoglin nor CD34 expression adequately reflects true MVD values in tumor tissues, and the use of Abs to both antigens may be necessary to achieve realistic results. Additional studies in larger patient cohorts with antibodies specific for all endothelial cells are to define the role of MVD as a predictive parameter in HNSCC and other malignancies.

## References

[1] Carvalho AL (2005). Trends in incidence and prognosis for head and neck cancer in the United States: a site-specific analysis of the SEER database. Int J Cancer.

[2] Folkman J (1971). Tumor angiogenesis: therapeutic implications. N Engl J Med.

[3] Weidner N (1992). Tumor angiogenesis: a new significant and independent prognostic indicator in early-stage breast carcinoma. J Natl Cancer Inst.

[4] Hollingsworth HC (1995). Tumor angiogenesis in advanced stage ovarian carcinoma. Am J Pathol.

[5] Fox SB, Harris AL (2004). Histological quantitation of tumour angiogenesis. APMIS.

[6] Perez-Gomez E (2010). The role of the TGF-beta coreceptor endoglin in cancer. Sci World J.

[7] Fonsatti E, Maio M (2004). Highlights on endoglin (CD105): from basic findings towards clinical applications in human cancer. J Transl Med.

[8] Dallas NA (2008). Endoglin (CD105): a marker of tumor vasculature and potential target for therapy. Clin Cancer Res.

[9] Weidner N (1991). Tumor angiogenesis and metastasis–correlation in invasive breast carcinoma. N Engl J Med.

[10] Uzzan B (2004). Microvessel density as a prognostic factor in women with breast cancer: a systematic review of the literature and meta-analysis. Cancer Res.

[11] Des Guetz G (2006). Microvessel density and VEGF expression are prognostic factors in colorectal cancer. Meta-analysis of the literature. Br J Cancer.

[12] Meert AP (2002). The role of microvessel density on the survival of patients with lung cancer: a systematic review of the literature with meta-analysis. Br J Cancer.

[13] Wang HL, Zhang ZL (2014). Analysis of the relationship between ultrasound of breast cancer DOT-SDI and the expression of MVD, VEGF and HIF-1alpha. Cell Biochem Biophys.

[14] Schimming R, Marme D (2002). Endoglin (CD105) expression in squamous cell carcinoma of the oral cavity. Head Neck.

[15] Kyzas PA, Agnantis NJ, Stefanou D (2006). Endoglin (CD105) as a prognostic factor in head and neck squamous cell carcinoma. Virchows Arch.

[16] Chien CY (2006). High expressions of CD105 and VEGF in early oral cancer predict potential cervical metastasis. J Surg Oncol.

[17] Chien CY (2006). Clinicopathologic significance of CD105 expression in squamous cell carcinoma of the hypopharynx. Head Neck.

[18] Marioni G (2011). Laryngeal carcinoma prognosis after postoperative radiotherapy correlates with CD105 expression, but not with angiogenin or EGFR expression. Eur Arch Otorhinolaryngol.

[19] Yu M (2014). Intratumoral vessel density as prognostic factors in head and neck squamous cell carcinoma: a meta-analysis of literature. Head Neck.

[20] Dunphy F (2002). Microvessel density in advanced head and neck squamous cell carcinoma before and after chemotherapy. Anticancer Res.

[21] Yao Y (2007). Endoglin (CD105) expression in angiogenesis of primary hepatocellular carcinomas: analysis using tissue microarrays and comparisons with CD34 and VEGF. Ann Clin Lab Sci.

[22] Chen B (2014). The prognostic implications of microvascular density and lymphatic vessel density in esophageal squamous cell carcinoma: comparative analysis between the traditional whole sections and the tissue microarray. Acta Histochem.

[23] Bodnar M, Każmierczak S, Marszałek W (2012). Ocena gęstości mikrounaczynienia (MVD) w raku płaskonabłonkowym krtani. Przegląd Lekarski.

[24] Zvrko E, Mikic A, Vuckovic L (2009). CD105 expression as a measure of microvessel density in supraglottic laryngeal squamous cell carcinoma. Eur Arch Otorhinolaryngol.

[25] Zvrko E, Mikic A, Vuckovic L (2010). Clinicopathologic significance of CD105-assessed microvessel density in glottic laryngeal squamous cell carcinoma. Auris Nasus Larynx.

[26] Martone T (2005). Prognostic relevance of CD105 + microvessel density in HNSCC patient outcome. Oral Oncol.

[27] Kyzas PA (2005). Prognostic significance of VEGF immunohistochemical expression and tumor angiogenesis in head and neck squamous cell carcinoma. J Cancer Res Clin Oncol.

[28] Liao B (2010). Macrophage migration inhibitory factor contributes angiogenesis by up-regulating IL-8 and correlates with poor prognosis of patients with primary nasopharyngeal carcinoma. J Surg Oncol.

[29] Shieh YS (2004). Role of angiogenic and non-angiogenic mechanisms in oral squamous cell carcinoma: correlation with histologic differentiation and tumor progression. J Oral Pathol Med.

[30] Zhuo X (2015). Expression and clinical significance of microvessel density and its association with TWIST in nasopharyngeal carcinoma. Int J Clin Exp Med.

